# Epidemiology of work-related injuries, musculoskeletal disorders and dermatitis among hospital food service workers in a tertiary hospital in Asia

**DOI:** 10.1186/s12995-024-00413-w

**Published:** 2024-05-17

**Authors:** Kenneth Bao Ren Leong, Qin Xiang Ng, Wee Hoe Gan, Wee Tong Ng, John Wah Lim

**Affiliations:** 1 Department of Occupational and Environmental Medicine, Singapore General Hospital, Singapore, Singapore; 2 Health Services Research Unit, Singapore General Hospital, Singapore, Singapore; 3 Saw Swee Hock School of Public Health, National University of Singapore and National University Health System, Singapore, Singapore

**Keywords:** Workplace, Occupational injury, Hospital care, Policy

## Abstract

**Background:**

Despite the relative importance, the prevalence of workplace safety and health issues in hospital food service workers is not well studied. This study describes the epidemiology of work-related injuries and occupational diseases among hospital food service workers (FSWs) in a tertiary hospital in Singapore.

**Methods:**

Using a total population sampling approach, a cross-sectional self-administered questionnaire was distributed to all FSWs employed at a major tertiary hospital in Singapore.

**Results:**

The response rate was 98.4% (*n* = 125). The overall prevalence of workplace injuries and musculoskeletal symptoms was 35% (*n* = 43) and 53% (*n* = 65) respectively. The most common workplace injuries were cuts/lacerations (35.8%), muscle strain (25.4%) and burns (19.4%). The prevalence of workplace injuries among staff performing food preparation duties was higher at 56.3% as compared to 21.6% among staff with no food preparation duties (*p* < 0.01). The prevalence of workplace injuries among staff performing cooking duties was also higher at 47.5%, compared to 29.3% among staff with no cooking duties (*p* = 0.05). Staff performing food preparation duties had a higher prevalence of musculoskeletal symptoms at 66.7% as compared to 44.6% among staff with no food preparation duties (*p* = 0.02). Obese staff had a higher prevalence of musculoskeletal symptoms at 78.9%, compared to overweight staff at 53.8% and staff with normal weight at 43.1% (*p* = 0.03).

**Conclusion:**

FSWs with jobs involving cooking and preparation of food, and those with obesity, are at higher risk of sustaining workplace injuries or musculoskeletal symptoms. Targeted interventions should be implemented for injury prevention and to mitigate these risks.

**Supplementary Information:**

The online version contains supplementary material available at 10.1186/s12995-024-00413-w.

## Introduction

The commitment towards enhancing standards related to workplace safety and health (WSH) within hospital settings is grounded in the fundamental principle that safeguarding the well-being of its workforce holds paramount significance, enabling hospitals to provide exceptional medical services to their patients. Over the passage of time, substantial emphasis has been directed towards ensuring the occupational safety and health of frontline healthcare personnel, encompassing professions like physicians and nurses. Nevertheless, the imperative of augmenting workplace safety and health conditions extends equivalently to encompass ancillary staff members, who contribute significantly to the holistic functioning of the hospital.

In particular, individuals employed within the domain of hospital food services encounter a diverse array of potential risks within their daily work routine. Beyond the prospect of occupational ailments, personnel working in hospital kitchen facilities are susceptible to a range of workplace-related injuries, encompassing instances such as burns, scalds, lacerations, and falls [1, 2].

A review of the literature regarding workplace injuries among food service workers found that most studies have typically focused on food service workers in restaurants and food stalls. In recent studies by Jahangiri et al. [3] and Abdelsalam et al. [4], work-related musculoskeletal symptoms, cuts, thermal burns as well as falls were among the most frequently reported occupational injuries. In an Irish study conducted by Gleeson [5], among food service workers in the catering industry, cuts and lacerations were found to be the most common injury, followed by burns and scalds. The epidemiology of workplace injuries appears to differ between food service workers across various settings. Data from the Bureau of Labour Statistics reported differences in the profile of injuries and illness between food service workers working in restaurants as compared to other settings, such as mobile food trucks and large-scale caterers [6].

Given that our survey period (which enquired exposures and symptoms in the past 12 months prior to January 2023) overlapped with the national movement restrictions, COVID-19 containment measures, as well as the Omicron wave in Singapore [7], it is also relevant to consider the potential impact of the pandemic and pandemic-related measures on occupational injuries. Apart from traditional hazards faced by food service workers, it is conceivable that the workers (especially those within healthcare settings) may experience increased workload from manpower shortages and increased stress and anxiety as a result of the lockdown measures [8]. Based on a study that analysed workers’ compensation data from 2019 to 2021, the authors found increased claims for stress or mental disorders during the pandemic but no increased likelihood of occupational injuries among workers (including food services staff) at nursing care facilities in California, United States [9].

Even outside of the COVID-19 pandemic, the prevalence of workplace safety and health issues in hospital food service workers is not well studied. Apart from a single retrospective study published in 2007 providing a descriptive analysis of workplace injury rates utilizing data from a surveillance database [10], there are no recent or detailed studies providing further insight into workplace health and safety issues faced by hospital food services workers. In the study by Alamgir et al. [10], musculoskeletal injuries were the most common, followed by percutaneous injury and contusions. The study also found a lower risk of workplace injury among older workers as compared to younger workers and a lower risk among males compared to females. Cooks were also found to be at higher risk of workplace injuries as compared to other food service workers [10].

It is expected that the working environment and job demands in restaurants and even commercial kitchens will differ from that of hospital food services. Hospital food services produce cooked food on a larger scale as the hospital kitchens are required to service the dietary requirements for all patients in the hospital. In Singapore General Hospital (SGH), the largest tertiary hospital providing acute care in Singapore, the hospital kitchen provides three meals a day for the entire inpatient population. With a bed capacity of more than a thousand beds [11], the kitchen is required to produce a few thousand meals per day. In addition, hospital food services have added logistical responsibilities, such as providing last mile delivery services so that each patient receives their meal at the designated meal periods.

Given the unique challenges and operational requirements in hospital food services, work processes in hospital kitchens may involve the use of machinery and robotics to improve the efficiency of food production. As such, the epidemiology of workplace health and safety issues in hospital food services is expected to differ as compared to that of regular restaurants and food stalls. Thus, it is important to study the epidemiology of workplace health and safety issues in hospitals food services to guide targeted interventions for these workers.

## Methods

This cross-sectional study is reported according to the STROBE (strengthening the reporting of observational studies in epidemiology) guidelines [12].

### Participants and procedure

This study employed a cross-sectional design, involving the distribution of a self-administered questionnaire to all food service workers employed at Singapore General Hospital, the largest tertiary hospital in Singapore. This data collection process spanned a 4-week duration from January 16, 2023, to February 10, 2023. The approach of total population sampling was utilized due to the well-defined and manageable size of the target population. The distribution of printed questionnaires took place through supervisors within the food services department. Workers who provided their consent to participate were instructed to place their completed questionnaires into designated secure collection boxes situated within the workplace premises. Subsequently, the study team transcribed and compiled the completed questionnaires, with the resulting data being stored safely and securely in the organisation’s research database.

### Questionnaire creation

Prior to questionnaire creation, a workplace assessment was conducted by the study team to understand the various work processes in the hospital kitchen to develop appropriate questions on work processes as well as occupational hazards in the study questionnaire.

After iterative team discussions and meetings, the finalized study questionnaire comprised of three parts. In the first part, demographic characteristics of food services workers in the department were collected. The second part collected data on the work processes in hospital kitchens while the third part focused on the prevalence of occupational diseases and work-related injuries.

Information on demographic and work processes were collected to identify risk factors related to work-related injuries and occupational diseases. Demographic variables collected include age group, gender, body mass index (BMI), job title and length of employment. Variables related to work processes included type of duties and tasks performed at work, such as Transfers and Lifting, Food Preparation, Cooking, Plating, Cleaning, Administrative and Management Duties.

To investigate the prevalence of symptoms suggestive of occupational diseases, such as work-related musculoskeletal disorders and occupational dermatitis, the questionnaire was constructed by the adaptation of existing validated questionnaires. The survey included the integration of the Nordic Musculoskeletal Questionnaire (NMQ) [13], which is widely employed to evaluate musculoskeletal symptoms. The NMQ is a standardized and validated 7-item survey that identifies symptoms based on specific body parts. Additionally, the survey integrated the Nordic Occupational Skin Questionnaire (NOSQ) [14], comprising inquiries aimed at gauging the prevalence of occupational skin disorders affecting the upper limbs.

For assessing the prevalence of workplace injuries, the survey also encompassed queries concerning the quantity and nature of such injuries over the course of the participant’s employment in the department. The types of workplace injuries were categorized as follows: (i) Cuts/Lacerations, (ii) Falls, (iii) Struck by Object, (iv) Burns, and (v) Musculoskeletal Strains.

The questionnaire was constructed in English and translated into Chinese and Malay languages to cater to the varying ethnicities of the food service workers, before being back-translated by an independent language teacher proficient in both languages and checked by the team for semantic equivalence. A copy of the questionnaire can be found in the Supplementary Material.

### Ethical approval

The research proposal was presented to the SingHealth Centralized Institutional Review Board (CIRB) and exempted from review as the study was part of a quality improvement and review process to improve the health of food service workers in hospital kitchens. Nonetheless, this study was performed in line with the principles of the Declaration of Helsinki. No directly identifiable personal data was collected and only aggregate data was reported. Data collected in the course of the study was handled with care and stored securely on an encrypted repository.

### Statistical analysis

All results were analyzed using STATA version 13 (Stata Corporation, TX). Descriptive statistics were presented for demographic variables, work process factors, as well as the prevalence of work-related injuries and occupational diseases. Tests for statistical significance, such as the chi-square test, fisher exact test, and t-test were used to compare differences in the prevalence of occupational disease and work-related injuries based on demographic and work-related factors. Logistic regression was performed to identify risk factors of work-related injuries and occupational diseases. In the regression model, BMI was analysed as a continuous variable to increase our sensitivity to detect subtle changes or associations with health outcomes [15]. The age categories 60–69 years and > 69 years were combined given their small cell counts, similarly, related job processes (admin and management, cleaning dishes and cleaning equipment, and plating and transportation of food) were combined for analysis as regression estimates based on small number of counts are less precise and more unstable. The level of significance was maintained at alpha of 0.05.

## Results

### Study demographics

The response rate was 98.4% (125 out of 127). Of the 125 responses, 3 questionnaires were incomplete and thus excluded from the analysis. As such, a total of 122 responses were retained for analysis.

In the study population, there were more males (66.4%) as compared to females (33.6%). A large proportion of the study population were of Chinese ethnicity (82.0%). There was an even age distribution of study participants aged from 20 to 69 years old, with a small proportion of staff aged > 69 years old (5.7%). The largest group of study participants comprised of chefs (35.9%), followed by dietary attendants (17.2%) (Table 1).

**Table 1:** Demographics of study respondents (*N* = 122)

Demographic variables	*n* (%)
**Gender**	
Female	41 (33.6)
Male	81 (66.4)
**Age (years)**	
20–29	17 (13.9)
30–39	29 (23.8)
40–49	24 (19.7)
50–59	22 (18.0)
60–69	23 (18.8)
> 69	7 (5.7)
**Ethnicity**	
Chinese	100 (82.0)
Indian	7 (5.7)
Malay	12 (9.8)
Others	3 (2.5)
**Years in workplace**	
0–5 years	57 (47.6)
> 5 years	65 (53.3)
**Job Title**	
Administration	6 (4.9)
Chef	45 (36.9)
Cleaner	13 (10.7)
Dietary Attendant	21 (17.2)
Dishwasher	15 (12.3)
Management	19 (15.6)
Plater	3 (2.4)
**BMI (kg/m** ^**2**^ **)**	
< 23	51 (41.8)
23–27.4	52 (42.6)
≥ 27.5	19 (15.6)

### Workplace injuries

The overall prevalence of workplace injuries in the study population was 35% (*n* = 43). A total of 67 workplace injuries were reported among 43 staff. The most common injuries were cuts/lacerations (*n* = 24, 35.8%), muscle strain (*n* = 17, 25.4%), burns (*n* = 13, 19.4%), falls (*n* = 7, 10.4%) and hit by object (*n* = 6, 9.0%) (Supplementary Table 1).

There were statistically significant differences in prevalence rates of workplace injuries among different groups of kitchen staff (Table 2). Chefs and dietary attendants had the highest prevalence of workplace injuries at 48.9% and 47.6% respectively. This finding corresponds to statistically significant differences in workplace injuries found based on work duties (Table 3). The prevalence of workplace injuries among staff performing cooking duties was higher at 47.5%, compared to 29.3% among staff who had no cooking duties (*p* = 0.048). The prevalence of workplace injuries among staff performing food preparation duties was also higher at 56.3% as compared to 21.6% among staff who had no food preparation duties (*p* < 0.01).

There were no statistically significant differences in prevalence rates of workplace injuries by gender, age, BMI, ethnicity, and work experience.

Having adjusted for age, gender, BMI and work processes in the logistic regression model (Table 4), we found that workers whose job involved cooking and preparing food had significantly increased likelihood of workplace injuries (OR 8.67, 95% CI: 1.47–51.22, *p* = 0.02) compared to other workers.

**Table 2:** Demographic characteristics of food service workers in hospital kitchens and prevalence of workplace injuries, musculoskeletal symptoms and dermatitis

Demographic variables	Prevalence of workplace injuries, % (*n*)	*P*-value	Prevalence of musculoskeletal symptoms, % (*n*)	*P*-value	Prevalence of dermatitis, % (*n*)	*P*-value
Gender						
Female	31.7 (13)	0.56	51.2 (21)	0.75	9.8 (4)	0.47
Male	37.0 (30)		54.3 (44)		12.2 (5)	
Age (years)						
20–29	41.2 (7)	0.40	58.8 (10)	0.45	11.8 (2)	0.12
30–39	44.8 (13)		69.0 (20)		17.2 (5)	
40–49	20.8 (5)		45.8 (11)		8.3 (2)	
50–59	36.4 (8)		45.5 (10)		0	
60–69	34.8 (8)		47.8 (11)		0	
≥ 69	14.3 (1)		42.9 (3)		0	
Ethnicity						
Chinese	37.0 (37)	0.24	55.0 (55)	0.19	9.0 (9)	0.54
Indian	0		14.3 (1)		0	
Malay	41.7 (5)		58.34 (7)		0	
Others	33.3 (1)		66.7 (2)		0	
Number of years worked in SGH Food Services						
0–5 years	33.3 (19)	0.68	54.4 (31)	0.82	7.0 (4)	0.89
> 5 years	36.9 (24)		52.3 (34)		7.7 (4)	
Job Title						
Administration	16.7 (1)	0.04	16.7 (1)	0.07	16.7 (1)	0.25
Chef	48.9 (22)		62.2 (28)		11.1 (5)	
Cleaner	7.7 (1)		38.5 (5)		0	
Dietary Attendant	47.6 (10)		52.4 (11)		0	
Dishwasher	20.0 (3)		33.3 (5)		0	
Management	31.5 (6)		73.7 (14)		15.8 (3)	
Plater	0		33.3 (1)		0	
BMI (kg/m^2^)						
< 23	37.3 (19)	0.33	43.1 (22)	0.03	7.8 (4)	0.65
23–27.4	28.8 (15)		53.8 (28)		5.8 (3)	
≥ 27.5	47.4 (9)		78.9 (15)		10.5 (2)	

**Table 3:** Work processes and prevalence of workplace injuries and musculoskeletal symptoms

Work-related factors	Prevalence of workplace injuries, % (*n*)	*P*-value	Prevalence of musculoskeletal symptoms, % (*n*)	*P*-value
Cooking Food				
No	29.3 (24)	0.05	48.8 (40)	0.15
Yes	47.5 (19)		62.5 (25)	
Preparation of Food				
No	21.6 (16)	< 0.01	44.6 (33)	0.02
Yes	56.3 (27)		66.7 (32)	
Transfer and Storage of Raw Food and Ingredients				
No	29.3 (22)	0.08	48.0 (36)	0.14
Yes	44.7 (21)		61.7 (29)	
Transportation of Cooked Food				
No	30.8 (24)	0.17	48.7 (38)	0.18
Yes	43.2 (19)		61.4 (27)	
Plating of Food				
No	33.8 (25)	0.68	50.0 (37)	0.37
Yes	37.5 (1)		58.3 (28)	
Clean Dishes				
No	40.5 (34)	0.07	54.8 (46)	0.63
Yes	23.7 (9)		50.0 (19)	
Clean Equipment				
No	40.5 (34)	0.40	54.9 (50)	0.53
Yes	29.0 (9)		48.4 (15)	
Admin				
No	37.9 (39)	0.16	53.4 (55)	0.95
Yes	21.1 (9)		52.6 (10)	
Management				
No	37.6 (38)	0.23	51.5 (52)	0.38
Yes	23.8 (5)		61.9 (13)	

**Table 4:** Logistic regression of selected variables with risk of workplace Injury and musculoskeletal symptoms

Variable	Workplace injury			Musculoskeletal symptoms		
	Odds ratio	95% CI	*P*-value	Odds ratio	95% CI	*P*-value
**Gender**						
Male	(ref)			(ref)		
Female	1.63	0.60–4.40	0.34	1.49	0.57–3.89	0.41
**Age (years)**						
20–29	(ref)			(ref)		
30–39	0.74	0.19–2.91	0.66	0.85	0.21–3.43	0.82
40–49	0.24	0.05–1.14	0.07	0.40	0.10–1.66	0.21
50–59	0.54	0.13–2.35	0.41	0.37	0.09–1.55	0.17
≥ 60	0.76	0.18–3.17	0.71	0.50	0.12–2.04	0.33
**BMI** ^**1**^	1.03	0.94–1.13	0.59	1.16	1.04–1.30	0.01
**Job Process**						
Admin and Management	(ref)			(ref)		
Cleaning Dishes and Equipment	2.39	0.33–17.32	0.39	1.48	0.30–7.36	0.63
Transfer and Storage of Raw Food and Ingredients	2.22	0.22–22.83	0.75	0.88	0.11–7.06	0.90
Cooking and Preparing Food	8.67	1.47–51.22	0.02	2.67	0.62–11.40	0.19
Plating and Transportation of Cooked Food	2.01	0.28–14.61	0.49	0.80	0.15–4.37	0.80

^1^BMI was treated as a continuous variable

### Musculoskeletal symptoms

The presence of musculoskeletal symptoms was defined as the presence of aches, pain or discomfort in any part of the body for the past 12 months. With this definition, the overall prevalence of musculoskeletal symptoms was 53% (*n* = 65). Among staff with musculoskeletal symptoms, the shoulders (34%), lower back (25%) and ankles/feet (28%) were the most common sites affected (full breakdown can be found in Supplementary Table 2).

Staff with BMI ≥ 27.5 were found to have a higher prevalence of musculoskeletal symptoms at 78.9% as compared to staff with BMI < 23 (43.1%) and staff with BMI of 23–27.4 (53.8%) (*p* = 0.03) (Table 2).

There were no statistically significant differences in prevalence of musculoskeletal symptoms found between the other demographic factors studied, although a higher rate of musculoskeletal symptoms was found in chefs (62.2%), dietary attendants (52.4%) and management staff (73.7%) (Table 2).

Among the work processes studied, staff who performed food preparation duties had a significantly higher prevalence of musculoskeletal symptoms at 66.7% as compared to 44.6% among staff who did not have food preparation duties (*p* = 0.02) (Table 3).

Similarly, in the logistic regression analysis, having adjusted for age, gender, BMI and work processes (Table 4), we found that workers with higher BMI were at higher risk of suffering from musculoskeletal symptoms (OR 1.16, 95% CI: 1.04–1.30, *p* = 0.01). Notably, workers whose job involved cooking and preparation of food were at higher risk of musculoskeletal symptoms (OR 2.67, 95% CI: 0.62–11.40, *p* = 0.19), compared to workers whose job involved administrative and management functions.

### Dermatological symptoms

The presence of upper limb dermatitis was defined as the presence of rashes across the hands, wrist or elbows at any time during the past 12 months. By this definition, the prevalence of hand and elbow dermatitis was 7.4% (*n* = 9) of which most occurred among chef and management staff (Table 2). Due to the low absolute numbers of dermatitis in this study population, subgroup analysis of dermatitis did not yield any significant findings.

## Discussion

Similar to studies involving food service workers in restaurants and food stalls, this study found that there was a high prevalence of work injuries and musculoskeletal symptoms among food service workers in a hospital’s food service department.

As compared to an earlier study conducted on cooks and food service workers in the healthcare sector in Canada in 2007 [10], we found a differing profile of workplace injuries among hospital food service workers at a major tertiary centre in Singapore. Our study found that the most common injuries sustained were cuts (35.8%), muscle strain (25.4%) and burns (19.4%). In the 2007 study, the most common workplace injuries were musculoskeletal strains (48.0%) followed by cuts (16.2%) and being hit by an object (12.7%) [10]. Besides geographic reasons, the differences observed could be due to shifting epidemiology of workplace injuries and changing workplace demands and job environments. A possible reason for the decrease in proportion of musculoskeletal strains could be the automation of many job processes, such as the use of Automated Guided Vehicles (AGVs) for food delivery, thus decreasing the need for physical handling of kitchen equipment and food. The use of AGVs for food transportation and delivery from the kitchens to the wards took place in Singapore progressively since 2015 and all public acute hospitals in Singapore have now adopted this technology.

Our study found that the prevalence of musculoskeletal symptoms among hospital food service workers was 53%, which is lower compared to other recent studies among food service workers. An Iranian study among restaurant workers found the prevalence to be higher at 70% [3]. While an Egyptian study found a prevalence of 90.6% among food service workers in student hostels [4]. Comparing to countries with similar socio-economic development, a Korean study among female meal service workers reported the prevalence of musculoskeletal symptoms to be 79.6% [16], which was also higher than the prevalence of symptoms found among female workers in our study, which was 51.2%. Without conducting workplace assessments in these other countries, it may be difficult to accurately account for the differences in rates of musculoskeletal symptoms. However, it could be hypothesized that the use of engineering controls to decrease the effort needed to physically handle food and raw ingredients in our hospital kitchen would be an important factor. Some of the engineering controls implemented in our local hospital kitchen include the use of trolleys to transport raw ingredients, the use of shelves of adequate height to prevent storage of raw ingredients on shelves that are too high as well as the use of cooking equipment that reduces the need for physically lifting or handling of food. In Singapore, such engineering controls were first implemented in large scale commercial kitchens. These technologies were found to be useful and subsequently introduced in the hospital kitchens.

In our study, chefs and dietary attendants were found to have higher rates of workplace injuries and musculoskeletal symptoms as compared to other groups of staff in food services. This correlated with our findings that job processes that directly involved cooking and food preparation are also associated with increased rates of work injuries and musculoskeletal symptoms as compared to those related to cleaning and transportation of food. This confirms the expectation that job processes contribute to the increased risk of workplace injuries and occupational diseases among chefs and dietary attendants.

Similar to existing studies in the food services industry [10, 17], older workers were found to be at lower risk of workplace injuries, as compared to younger workers. A possible reason for this finding is that older workers have more experience in the work processes and the hazards at work. This could also be attributed to the healthy worker effect as this is a cross-sectional study studying prevalence and older workers who suffer from workplace injuries may experience greater morbidity which impedes their ability to continue working.

Using the WHO Asian BMI classification [18], our study found that persons who are obese (BMI ≥ 27.5) had a higher prevalence of musculoskeletal symptoms as compared to healthy or overweight individuals. This finding is consistent with other studies which also found similar associations between BMI and musculoskeletal symptoms in the broader working population [19]. This could be attributed to poorer postural stability and poorer control of body movements in obese persons, leading to greater strain on the musculoskeletal systems of obese individuals [20, 21]. Interestingly, it was also found that a higher BMI was not correlated with a higher risk of workplace injuries, supporting findings from existing literature [22]. This could be hypothesised to be due to the ability of the individual to accommodate to the hazards posed by obesity as changes in body habitus and function occur slowly over time in obesity.

The prevalence of upper limb dermatitis reported in our study was low at 7.4%. This was slightly lower when compared to a local study conducted in restaurant, catering, and fast-food outlets in Singapore in 2009, which found the prevalence to be 10% [23]. As the need to perform wet work has been thought to be a risk factor for dermatitis among food service workers [24], two reasons for the lower prevalence in our study could be due to the use of kitchen machinery and gloves for washing of vegetables, fruits and cutlery, which reduces the exposure to wet work in our setting.

### Strengths and limitations

Our study had several notable strengths and limitations. This is the pioneer study in Southeast Asia and one of the few in the world to examine the epidemiology of work-related injuries and occupational diseases among hospital food service workers. This is a group of workers that has been largely neglected in modern occupational medicine and research. The study also had a sizeable sample and benefited from a high response rate among staff, which reduces the impact of selection bias. While we acknowledge the concern that the high participation rate could suggest undue coercion, we believe it is instead indicative of the employees’ genuine interest and engagement in topics related to workplace safety and health, especially since they work in a healthcare setting. Another significant strength of the study is the incorporation of validated questionnaires to assess the prevalence of musculoskeletal and dermatological conditions, which reduces the likelihood of information bias.

Nonetheless, our study had several limitations that should be acknowledged. Firstly, it was conducted at a single site, which may restrict the generalizability of the findings. Despite sampling the entire food service worker population within the hospital, the overall study size remained relatively small, especially for the number of analyses performed. Secondly, our study depended on self-reported data provided by the study participants, rendering it vulnerable to information biases, including recall bias and social desirability bias. However, social desirability bias was in part mitigated as this was an anonymous survey. For future study, collecting further information on job exposures (e.g., the number of hours worked, list of tasks completed, safety behaviours and personal protective equipment (PPE) use) would make findings from the Nordic questionnaires more useful. Similarly, further information on work injuries would have provided a more robust discussion, and other specific risk factors in the workplace could be studied as well.

## Conclusions

This study contributes to the limited pool of existing studies by describing the epidemiology of work-related injuries and occupational diseases among hospital food service workers in a major tertiary hospital in Singapore. Specific groups of staff in hospital kitchens, such as persons with job demands involving cooking and preparation of food, as well as those who are obese are at higher risk of sustaining workplace injuries or musculoskeletal symptoms. Targeted interventions should continually be implemented to mitigate these risks.

### Electronic supplementary material

Below is the link to the electronic supplementary material.

Supplementary Material 1

## Data Availability

Due to restrictions of the institutional review board, the datasets used and/or analysed during the current study are only available from the corresponding author on reasonable request.

## Declarations

### Patient and public involvement

Patients and/or the public were not involved in the design, or conduct, or reporting, or dissemination plans of this research.
