# Supplementary material for: Epidemiology of work-related injuries, musculoskeletal disorders and dermatitis among hospital food service workers in a tertiary hospital in Asia
Source: J Occup Med Toxicol. 2024 May 17;19:18. doi: 10.1186/s12995-024-00413-w (PMC11100083; doi:10.1186/s12995-024-00413-w)
Supplement: Supplementary file 1 — Supplementary Material 1 [file 12995_2024_413_MOESM1_ESM.docx]

**Supplementary Material**

**Table S1.** Copy of survey questionnaire in English

| **PART A: DEMOGRAPHICS** | | | |
| --- | --- | --- | --- |
| **1. Gender**  Male  Female | **2. Ethnicity**  Chinese  Malay  Indian  Others: _____________ | | |
| **3. Age**  < 20 years old  20 – 29 years old  30 – 39 years old  40 – 49 years old  50 – 59 years old  60 – 69 years old  > 69 years old | | | |
| **4. Number of Years Worked in SGH Food** **Services**: ______ years | | | |
| **5. Job Title**  Chef/Cook  Plater  Dishwasher  Cleaner  Dietary Attendant  Administration  (Store)  Dietary Attendant  Management  (Operations Supports) | | **6. Height (cm): ______________**  **Weight (kg): ______________** | |
| **PART B: WORK DETAILS** | | |  |
| **7. Duties Performed in Food Services**  *(select all that applies)*:  Transfer and lifting of raw food and ingredients (e.g. receiving and storing of food in storeroom, transfer of food from storeroom to kitchen, pushing and pulling of loaded trolleys, lifting baskets of vegetables, fruits and canned food)  Preparation of food (measuring amount of food and condiment, cutting of ingredients, washing ingredients, mixing of ingredients)  Cooking of food  Plating of food  Transportation of cooked food (pushing and pulling of food trolleys)  Cleaning of utensils and dishes  Cleaning of kitchen equipment  Administrative duties  Management and supervisory duties  Others (please specify): _______________________ | | |  |

| **PART C: PREVALENCE OF OCCUPATIONAL DISEASES AND WORKPLACE INJURIES** | | | | |
| --- | --- | --- | --- | --- |
| The picture below shows the approximate position of the parts of the body referred to in the questionnaire. Limits are not sharply defined, and certain parts overlap. You should decide for yourself in which part (including left sided, right sided or both for certain body parts) you have or have had trouble in.  . **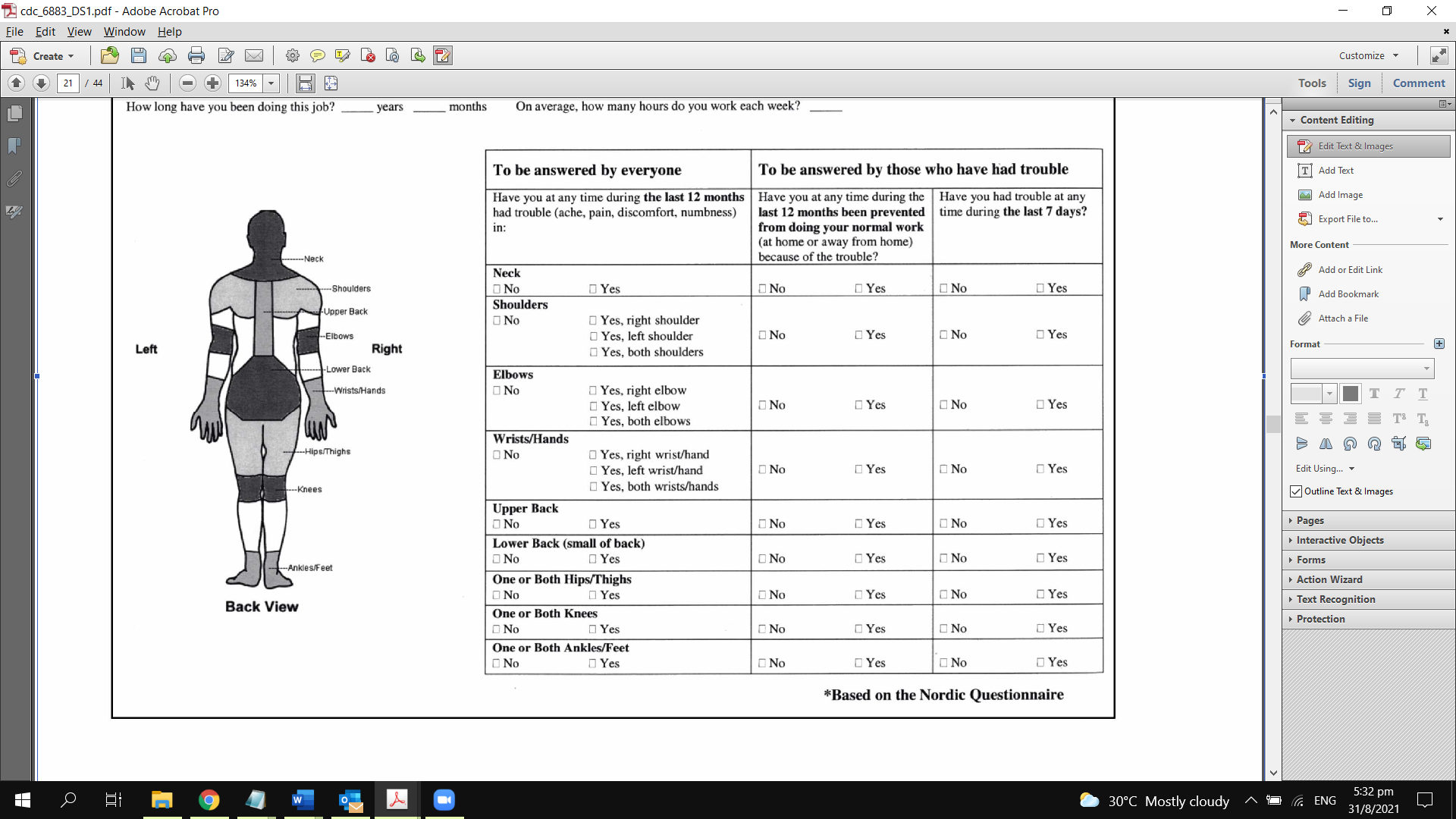** | | | | |
|  | **8. Have you, at any time during the last 12 months, had trouble (such as ache, pain, discomfort, numbness) in:** | | **9. During the last 12 months, have you been prevented from carrying out normal activities (e.g. housework, hobbies) because of trouble in:** | **10. During the last 7 days, have you had trouble (such as ache, pain, discomfort, numbness) in:** |
| **Neck** | Yes | No | Yes  No | Yes  No |
| **Shoulder** | Yes, right side  Yes, left side  Yes, both sides | No | Yes  No | Yes  No |
| **Upper back** | Yes | No | Yes  No | Yes  No |
| **Elbows** | Yes, right side  Yes, left side  Yes, both sides | No | Yes  No | Yes  No |
| **Wrists/**  **Hand** | Yes, right side  Yes, left side  Yes, both sides | No | Yes  No | Yes  No |
| **Lower back** | Yes | No | Yes  No | Yes  No |
| **Hips/Thigh** | Yes | No | Yes  No | Yes  No |
| **Knees** | Yes | No | Yes  No | Yes  No |
| **Ankles/Feet** | Yes | No | Yes  No | Yes  No |
| **11. Have you ever had hand eczema?**  Yes  No **(Skip Q12,13,14,15)** | | | | |
| **12. When did you last have eczema on your hands?**   1. I have it right now 2. not right now but within the past 3 months 3. between 3-12 months ago 4. more than 12 months ago   In which year was the last time? _________ (year) | | | | |
| **13. Have you noticed that contact with certain materials, chemicals or anything else  in your work makes your hand eczema worse?**  Yes, please specify _________________  No  Don’t know | | | | |
| **14. Have you noticed that contact with certain materials, chemicals or anything else  outside your work makes your hand eczema worse?**  Yes, please specify _________________  No  Don’t know | | | | |
| **15. Does your hand eczema improve when you are away from your normal work?**  No  Yes, sometimes  Yes, usually  Don’t know | | | | |
| **16. Have you ever had eczema on your wrists or forearms (excluding fronts of elbows)?**  Yes  No **(Skip Q17, Q18, Q19 and Q20)** | | | | |
| **17. When did you last have eczema on your wrists/forearms?**   1. I have it right now 2. not right now but within the past 3 months 3. between 3-12 months ago 4. more than 12 months ago   In which year was the last time? _________ (year**)** | | | | |
| **18. Have you noticed that contact with certain materials, chemicals or anything else  in your work makes your wrist/forearm eczema worse?**  Yes, please specify _________________  No  Don’t know | | | | |
| **19. Have you noticed that contact with certain materials, chemicals or anything else  outside your work makes your wrist/forearm eczema worse?**  Yes, please specify _________________  No  Don’t know | | | | |
| **20. Does your wrist/forearm eczema improve when you are away from your normal work?**  No  Yes, sometimes  Yes, usually  Don’t know | | | | |
| **21. Have you ever had an itchy rash that has been coming and going for at least 6 months,  and at some time has affected skin creases?**  *(skin creases include folds of elbows, behind the knees, fronts of ankles, under buttocks, around the neck, ears, or eyes)*  Yes,  No  Don’t know | | | | |
| **22. I have suffered an injury in my current workplace before:**  No  Yes  If Yes, what type of injury:  *(select all that applies)*  Cut/Laceration  Burn/Scald  Falls  Hit by object  Muscle Strain  Other (please specify): _______________________ | | | | |

**Table S2.** Type of work injuries sustained (N=67)

| **Type of injuries** | **n (%)** |
| --- | --- |
| Cut/Laceration | 24 (35.8) |
| Falls | 7 (10.4) |
| Muscle Strain | 17 (25.4) |
| Hit by Object | 6 (9.0) |
| Burns | 13 (19.4) |

**Table S3.** Prevalence of musculoskeletal symptoms, regional (N=122)

| **Body region** | **%** |
| --- | --- |
| Neck | 23 |
| Shoulder | 34 |
| Upper Back | 10 |
| Elbow | 12 |
| Wrist & Hand | 22 |
| Lower Back | 25 |
| Hip | 8 |
| Knee | 20 |
| Ankle & Feet | 28 |
